# Lagrangian coherent structure assisted path planning for transoceanic autonomous underwater vehicle missions

**DOI:** 10.1038/s41598-018-23028-8

**Published:** 2018-03-15

**Authors:** A. G. Ramos, V. J. García-Garrido, A. M. Mancho, S. Wiggins, J. Coca, S. Glenn, O. Schofield, J. Kohut, D. Aragon, J. Kerfoot, T. Haskins, T. Miles, C. Haldeman, N. Strandskov, B. Allsup, C. Jones, J. Shapiro

**Affiliations:** 1 División de Robótica y Oceanografía Computacional (IUSIANI), Universidad de Las Palmas de Gran Canaria, Las Palmas de Gran Canaria, Spain; 2 Instituto de Ciencias Matemáticas, CSIC-UAM-UC3M-UCM, C/Nicolás Cabrera 15, Campus de Cantoblanco UAM, 28049 Madrid, Spain; 3 U.D. Matemáticas, Universidad de Alcalá, 28871 Alcalá de Henares, Spain; 4 School of Mathematics, University of Bristol, Bristol, BS8 1TW United Kingdom; 5 Rutgers University Center of Ocean Observing Leadership, School of Environmental and Biological Sciences, Rutgers University, New Brunswick, NJ 08901 USA; 6 Teledyne Webb Research, North Falmouth, MA 02566 USA

## Abstract

Transoceanic Gliders are Autonomous Underwater Vehicles (AUVs) for which there is a developing and expanding range of applications in open-seas research, technology and underwater clean transport. Mature glider autonomy, operating depth (0–1000 meters) and low energy consumption without a CO_2_ footprint enable evolutionary access across ocean basins. Pursuant to the first successful transatlantic glider crossing in December 2009, the Challenger Mission has opened the door to long-term, long-distance routine transoceanic AUV missions. These vehicles, which glide through the water column between 0 and 1000 meters depth, are highly sensitive to the ocean current field. Consequently, it is essential to exploit the complex space-time structure of the ocean current field in order to plan a path that optimizes scientific payoff and navigation efficiency. This letter demonstrates the capability of dynamical system theory for achieving this goal by realizing the real-time navigation strategy for the transoceanic AUV named Silbo, which is a Slocum deep-glider (0–1000 m), that crossed the North Atlantic from April 2016 to March 2017. Path planning in real time based on this approach has facilitated an impressive speed up of the AUV to unprecedented velocities resulting in major battery savings on the mission, offering the potential for routine transoceanic long duration missions.

## Introduction

**Figure 1:**
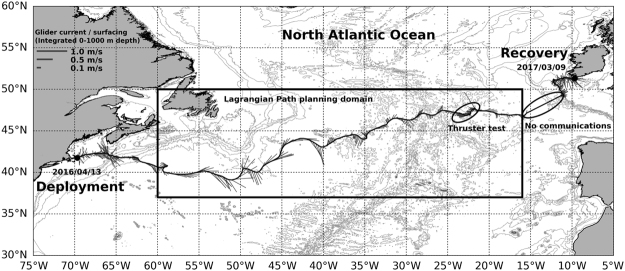
Silbo NE Atlantic crossing path. Silbo was deployed in Massachusetts on the 13th April 2016 and was recovered at the South of Ireland on the 9th March 2017 after completing a transect between 0–1000 meters depth of 6506.8 km in 330 days. The figure was created using python 3.5.2, matplotlib^36^ module 1.5.1 (https://www.python.org/downloads/release/python-352/). Bathymetry data was obtained from Gebco^37^ 2014 30 arc-second grid (http://www.gebco.net). Glider track and currents were derived from glider log navigation files. Coastlines were obtained from GSHHG - A Global Self-consistent, Hierarchical, High-resolution Geography Database (https://www.ngdc.noaa.gov/mgg/shorelines/gshhs.html).

Silbo, a deep Slocum glider in the Challenger mission was deployed in Massachusetts on the 13th April 2016 and was recovered at the South of Ireland on March 9th 2017. He completed 6506.8 km, gliding across the North Atlantic by following a saw tooth trajectory (see Fig. 1) through the top 1000 meters of the water column in 330 days by consuming 1.5 A ⋅ h/*day* (or 22.5 W ⋅ h/*day* at 15 V) from its lithium batteries (see https://marine.rutgers.edu/cool/auvs/index.php?gid=46).

Silbo’s flight demonstrated that autonomous underwater deep gliders will play a preeminent role in transoceanic ocean observation in coming years^1–3^. Expectations for transoceanic gliders are high due to their ability to map and monitor the marine environment without requiring direct human control. For this reason, they provide opportunities for data acquisition in areas of the ocean otherwise difficult, dangerous or impossible to access, including areas beneath tropical cyclones or ice sheets in polar regions^3,4^. Glider’s generate propulsion by modulating their buoyancy at specified depths (shallow or deep glider) and transferring a component of the induced vertical acceleration forward by means of a lifting body and swept wings. They are designed to have long endurance (months, years) and to navigate autonomously, being controlled by periodically surfacing for GPS fixes, data telemetry and opportunity for shore side operators to update the vehicle’s mission. One operational consequence of designing for endurance is that the effective but low horizontal speed (0.2–0.4 m/s) makes them extremely sensitive to the current fields that they experience. As underactuated vehicles, gliders are not necessarily capable of following an arbitrary trajectory to reach a required location. In this context, it would be a significant advantage to utilize the ocean current field in a way that could optimize the mission of the glider.

Typical conventional glider path planning methodologies for determining optimal paths have demonstrated their effectiveness in regional environments. Among these methodologies are linear programming, probabilistic sampling, potential fields or genetic algorithms and artificial intelligence methods such as A^*^ (see^5–11^). Some of these methods require the use of Regional Ocean Models (ROMs) forecast datasets with high space-time resolution (1/32^*o*^, hourly). Nowadays in the open ocean only low space-time resolution models are available (1/12^*o*^, daily) and therefore these techniques are not implementable in transoceanic glider crossing missions. In this regard if global models in the future would increase their resolution and accuracy it could be that regional methods provide efficient solutions also in these missions, but this is not the case for the current state of the art. Some attempts in this direction are references^12,13^ that describe the path planning A* technique used at the end of the first Atlantic glider crossing, once it approached the Iberian coast. In particular the method was implemented with the ROM ESEOO Iberian domain data (1/32°, hourly, +72 h). Additional regional path planning requirements, as for instance the demand of environmental obstacles by the Theta algorithm, are not available at global scale, or the need of a rather stable environment just subjected to small perturbations, a must for incremental methods such as D* and Phi*, does not work in a highly dynamic and changing environment like that found in the open ocean. Long-term long-distance path planning missions require guiding techniques that are useful for highly dynamic open-sea areas, and thus they must be based on robust and fundamental ocean features.

Transoceanic Slocum glider missions are relatively recent and until now there has been only a few of them. The first successful North East transatlantic mission was achieved by the Scarlet Knight RU27 glider in 2009^1^. This mission was preceded in 2008 by the RU17 glider attempt, which was unfortunately lost just off the coast of the Azores. Other subsequent missions have been performed by Cook, Drake, Silbo and RU29 gliders^2^. Missions have been an adventurous path to learning about a completely unexplored terrain and to gain information about many different aspects of the missions, ranging from glider flight dynamics, battery consumption, resets, bathymetry risks, aborts, piloting error, physical and biological impediments (such as barnacles adhesion and fouling) and their effects on long term navigation, etc. In the Silbo mission described in this article, navigation has been in the 0–1000 m depth range, however the 2009 Scarlet Knight RU 27 flew between 0–200 meters depth range and this allowed taking a maximum advantage of the Gulf stream speed, aligned with the direction of the voyage. In other missions, with gliders Drake and Cook, deep flying has been shown to be an effective way to fight unfavorable currents since at large depths currents are weaker. Since the first missions, in order to gain insights into the ocean landscape, different approaches have been considered. The first missions were flown using Sea Surface Temperature (SST) fields as a primary reference for mesoscale flow features, and waypoints were programmed according to the displayed structures. SST was chosen for its global availability, its relatively fast update cycle from AVHRR satellite data, and its ability to resolve many surface flow features. Alternatives to this product have been currents derived from the Sea Level Anomaly, 3D current fields from models, etc. In this letter, we demonstrate the success and promise of a new approach to path planning for future AUV crossing missions that was implemented for Silbo. This is the dynamical systems approach to transport that involves using the space-time structure of the ocean current field in a way that optimizes the propulsion of the glider in a manner that promotes sustainable missions. More specifically, the methodology proposed in this mission for supporting the waypoint selection uses Lagrangian Coherent Structures (LCS). This is not disconnected from velocity fields, but based on them since LCS provide a time dependent Lagrangian pattern (i.e. based on fluid particle trajectories) which at each day encompasses information from the velocity field in past and future days, and therefore is suitable for advising about Lagrangian paths, such as those followed by gliders. Eulerian velocity fields or instantaneous temperature fields used in previous missions are more rudimentary in this regard.

The idea of exploiting natural dynamics for vehicle transport has been previously used in space mission design. The work is similar in spirit to our work in the ocean in the sense that the gravitational field of the planetary system is used to determine a desired mission trajectory for a spacecraft with low thrust capabilities^14,15^. These ideas have also been previously proposed in oceanic setting, for planning glider routes through ocean currents^16^, but they have not been applied to transoceanic missions in the way that we have done for the Silbo mission.

## Results

Silbo’s control mechanisms allow the glider to control its heading so as to pass through manually defined waypoints (WPs) with or without compensating for local depth average current. Our goal is to extract information from the oceanic currents, in particular, about the natural dynamics of particle trajectories advected by ocean currents, since we expect that this knowledge will inform the choice of WPs. In the ocean, particles follow trajectories **x**(*t*) that evolve according to the dynamical system:

1 $$\frac{d{\bf{x}}}{dt}={\bf{v}}({\bf{x}}(t),t),$$

where **v**(**x**, *t*) is the velocity field of the ocean in the region of interest. In our analysis we will assume that the motion of particles is mainly horizontal. Many LCS studies have been performed in a two-dimensiolnal scenario in which is assumed that fluid parcels remain on surfaces of constant density (isopycnals), which are quasi-horizontal^17–22^. We will discuss deviations from horizontal motion afterwards.

**Figure 2:**
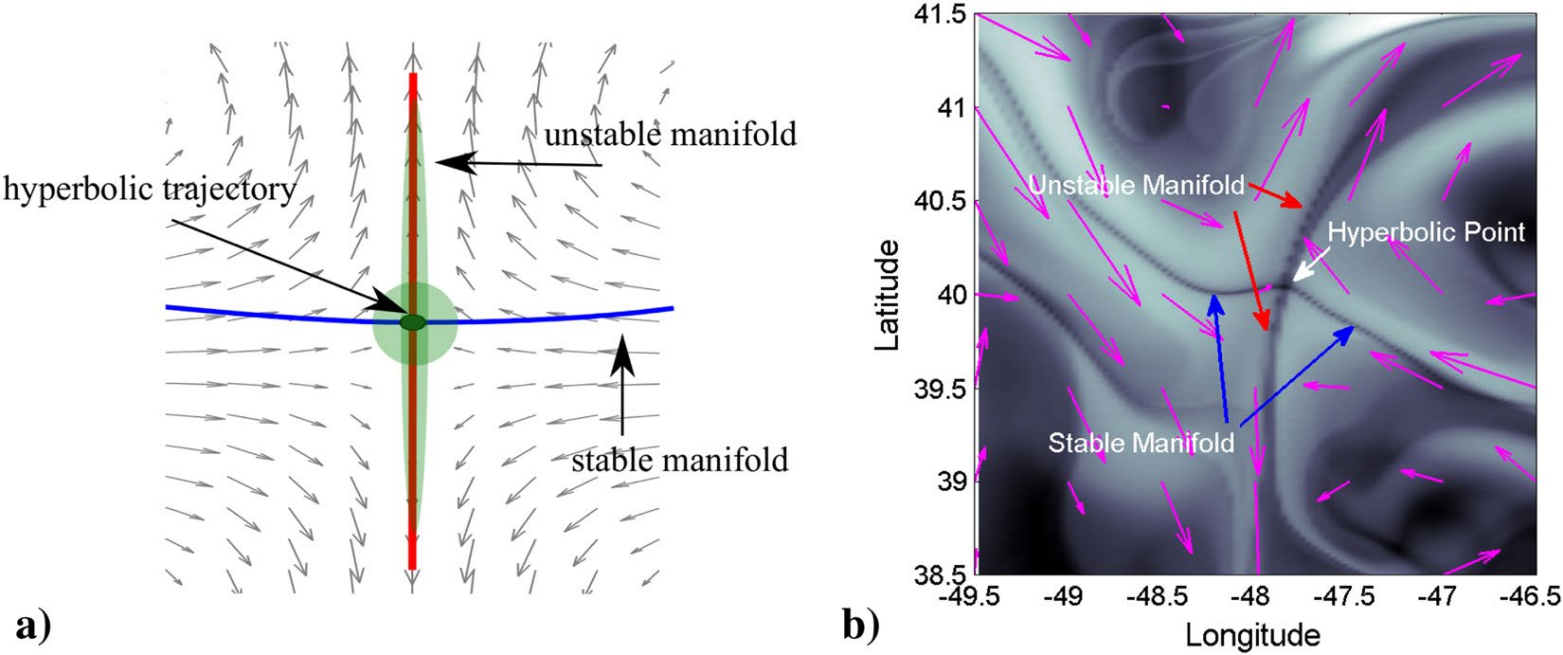
(**a**) A hyperbolic trajectory in a vector field. Particles at successive times evolve by approaching the hyperbolic point along the stable direction (blue) and getting away from it along the unstable direction (red). Green blobs illustrate this behavior. (**b**) Visualization of a hyperbolic point by means of the function *M* evaluated on Copernicus data on the 17th June 2016. The current field is drawn with magenta arrows.

A challenge here is that even flows with smooth velocity fields may exhibit complex particle trajectories. An approach for exploiting this complexity derives from the methodology of nonlinear dynamical systems theory. Rather than seeking to understand the behavior of large ensembles of particle trajectories, this approach is based on finding geometrical structures, known as Lagrangian Coherent Structures (LCSs) that divide the ocean into regions corresponding to qualitatively distinct particle motions^23–25^. The boundaries or barriers between these regions are time dependent material surfaces (which, mathematically, are invariant manifolds). This spatio-temporal template can be constructed with a recent technique referred to as Lagrangian Descriptors (LDs). The particular LD that we use is a function referred to as *M*^26–28^ which is defined as follows:

2 $$M({{\bf{x}}}_{0},{t}_{0},\tau )={\int }_{{t}_{0}-\tau }^{{t}_{0}+\tau }\Vert {\rm{v}}({\bf{x}}(t;{{\bf{x}}}_{0}),t)\Vert \,dt,$$

where $\parallel \cdot \parallel$ stands for the modulus of the velocity vector. At a given time *t*_0_, function *M*(**x**_0_, *t*_0_, *τ*) measures the arclength of trajectories starting at **x**(*t*_0_) = **x**_0_ as they evolve forwards and backwards in time for a time interval *τ*. Large *M* values, represented in white color (see Fig. 2), are related to regions of high speed fluid (such as straight or circular jets), while dark colors denote calm regions. One expects that large *M* values will favor glider propulsion, as far as the commanded-glider trajectory is aligned with the current, and that calm ocean regions will be related to slower glider motions.

The pattern displayed by the function *M* depends on *τ*. For small *τ*, the function *M* has a smooth output, while as the parameter *τ* is increased, sharp features and structures emerge highlighting LCS. Typically patterns provided by very large *τ* values reveal a more detailed description of the dynamical history of the system. In our setting we use data from the Global Ocean Model provided by Copernicus that has forecasts for 10 days, and thus this value fixes the operational upper threshold for the forward time integration period. In practice the integration period necessary to display the required LCS depends on the characteristics of each velocity field. We have verified that *τ* = 8 days is a sufficient choice for our data, and from the physical point of view this is consistent with the time required by the glider to navigate distances equivalent to the size of mesoscale ocean structures. In this way when the function *M* is computed for this sufficiently large *τ* it provides a detailed landscape from which it is possible to relate glider accelerations directly to the topography of this landscape. Of particular interest are features highlighting hyperbolic trajectories that are responsible for deflecting the grider’s trajectory. Regions in the fluid characterized by high expansion and contraction rates generate the stable and unstable structures in the flow field that characterize hyperbolic trajectories. Figure 2a illustrates how blobs in the neighborhood of these trajectories evolve, contracting along the stable direction and expanding along the unstable direction. Curves associated with the stable and unstable directions of these hyperbolic trajectories are referred to as stable and unstable manifolds and they indicate optimal paths for approaching and leaving the vicinity of these trajectories. Hyperbolic trajectories are recognizable in the pattern of *M* as the crossing points of singular features that highlight stable and unstable manifolds. For instance, an evaluation of *M* on a typical data set used in this study for *τ* = 8 days on the 17 June 2016 is displayed in Fig. 2b. Blue arrows mark the position of a stable manifold along which particles approach the hyperbolic trajectory at high speed and red arrows mark the position of an unstable manifold along which particles move away from the hyperbolic trajectory at high speed. Fluid particles slow their motion in the neighborhood of the hyperbolic trajectory. Magenta arrows representing the velocity field overlapped with the *M* pattern supports this interpretation of the stable and unstable directions. Next we describe how these effects, which are observed in the natural dynamics of particle trajectories advected by ocean currents, are also observed along the glider path. Given that hyperbolic points are objects for which there exist optimal pathways, they are a natural choice to be used as WPs for glider guidance. We describe next how this choice has proven to be effective.

The success of the described approach depends on how well the available velocity data represents the ocean state in the area in the domain of operation. The assessment of the ocean data with Lagrangian tools has been addressed in recent studies^20–22^. In particular^22^, shows the success of the Copernicus Marine Environment Monitoring Service (CMEMS) data (available at http://marine.copernicus.eu/) for monitoring oil spill events in real time, thus supporting this product as reliably representing ocean transport and confirming its high quality. Our study supports those findings, since the Copernicus Global Ocean and Iberia-Biscay-Ireland sea models have provided data which successfully supported the guidance of Silbo.

During the Trans-Atlantic mission, Silbo navigated at depths ranging from the surface to 900 meters following a saw tooth trajectory. This means that during navigation Silbo experienced currents at different depths from the upper Atlantic layers. Fully 3D studies performed in quasi 2D flows such as the ocean or the stratosphere have shown that 3D Lagrangian structures are close to those obtained by a’vertical extension’ of the evolving structures calculated in the 2D plane approximation^19,29,30^. Across most of this water column, Lagrangian patterns have a vertical curtain-like structure with only slight differences in each horizontal plane. Our approach to this problem then has been to study the 2D problem in Equation (1), by means of a representative 2D velocity field of the upper layers. To this end we have considered vertical averages of the instantaneous horizontal velocities components supplied by the model in the range 0–902 meters. We have compared these results with those obtained just by considering velocities at the 453 meters sigma layer, which is the mid layer of the total vertical range swept by the glider, and also averaged velocities over the water column 0–453 meters. We have found that Lagrangian structures are very similar in all cases, and we proceed to report results mainly with the first choice, i.e. averages across the range 0–902 meters. This choice is also supported by our observational experience as agrees well with the glider derived current field. Additional results with the other choices are also reported for comparative purposes.

**Figure 3:**
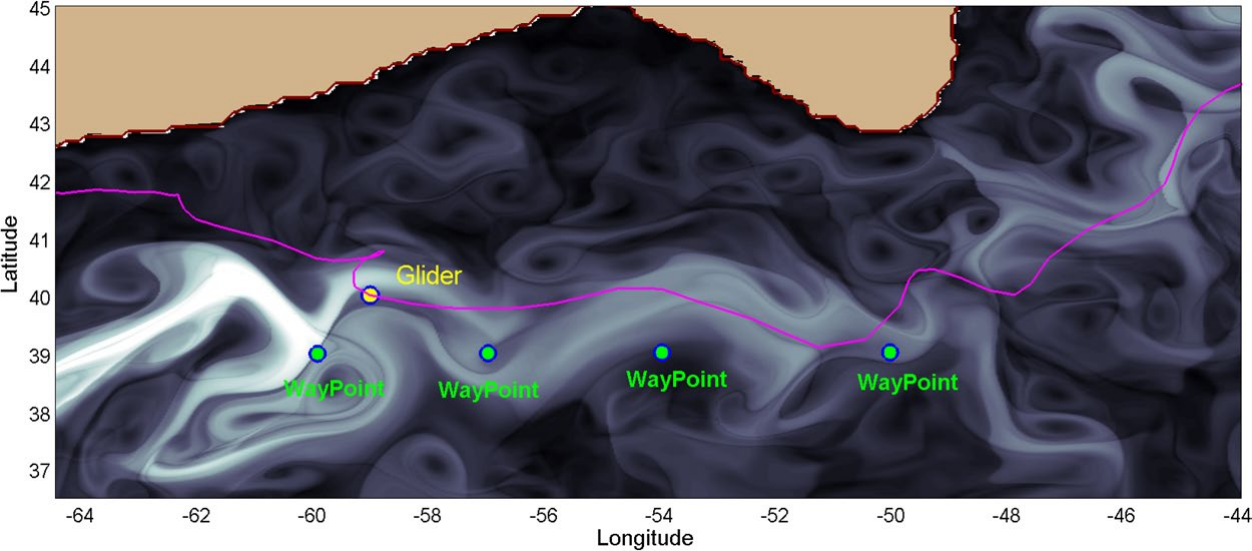
Lagrangian structures on the 30th May 2016 at 12:00 UTC in the NW Atlantic highlighted by the *M* function for *τ* = 8 days from CMEMS velocities averaged across depths 0–902 m. The glider Lagrangian path planning panel shows WPs used to cross the Gulf Stream (27th May–27th July 2016) (see video S1). This figure was created with MATLAB version R2010b (https://es.mathworks.com). The map shown is generated with a mask of values included in the CMEMS velocity field dataset. This mask indicates regions which correspond to continental shelf and sea.

**Figure 4:**
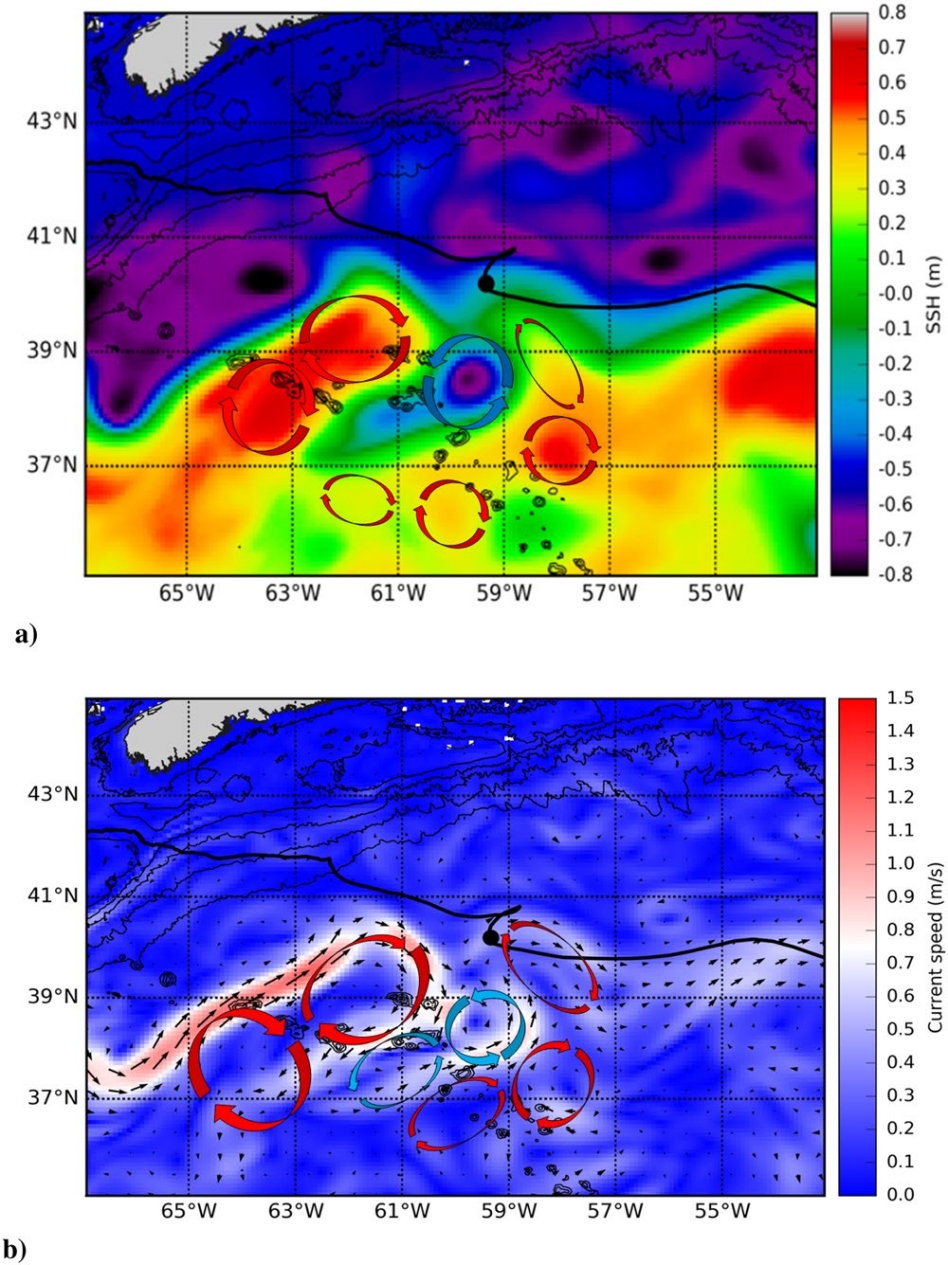
Glider path and Eulerian Gulf Stream description with the CMEMS dataset illustrating warm and cold vortex cores on the 30th May 2016. (**a**) Sea surface height (SSH) above geoid. (**b**) Velocity fields and averaged current speed (0–902 m). The figure was created using python 3.5.2, matplotlib^36^ module 1.5.1 (https://www.python.org/downloads/release/python-352/). Bathymetry data was obtained from Gebco^37^ 2014 30 arc-second grid (http://www.gebco.net). Glider track was extracted from glider log navigation files. Coastlines were obtained from GSHHG - A Global Self-consistent, Hierarchical, High-resolution Geography Database (https://www.ngdc.noaa.gov/mgg/shorelines/gshhs.html). SSH and current fields were obtained from Operational Mercator global ocean analysis and forecast (GLOBAL_ANALYSIS_FORECAST_PHY_001_024 product in Copernicus marine segment, http://marine.copernicus.eu).

Figure 3 shows the operational panel used for glider path planning. The integration period for these patterns is *τ* = 8 days. Waypoints are introduced according to the hyperbolic trajectories observed in Lagrangian patterns highlighted in the background, by looking for favorable navigation routes between hyperbolic trajectories towards the final destination of the glider. Stable and unstable manifolds associated to the hyperbolic trajectories are recognised as singular lines in the background. Hyperbolic trajectories are suitable to select as waypoints supporting glider bearing since these objects have invariant manifolds which provide an optimal path for reaching the WPs. Eulerian fields shown in Fig. 4 do not display such objects and therefore it is more difficult to find a criterion for fixing waypoints using solely Eulerian information.

The major findings of this work are summarized in the movie S1, which runs from the 15th April 2016 until the 1st November 2016. This animation overlaps the Lagrangian pattern provided by function *M* (obtained from velocities averaged in the 0–902 m range), with Silbo’s speed at different points along the glider path. Additionally, the movie displays instantaneous averaged velocity fields and the waypoint positions at different times.

**Figure 5:**
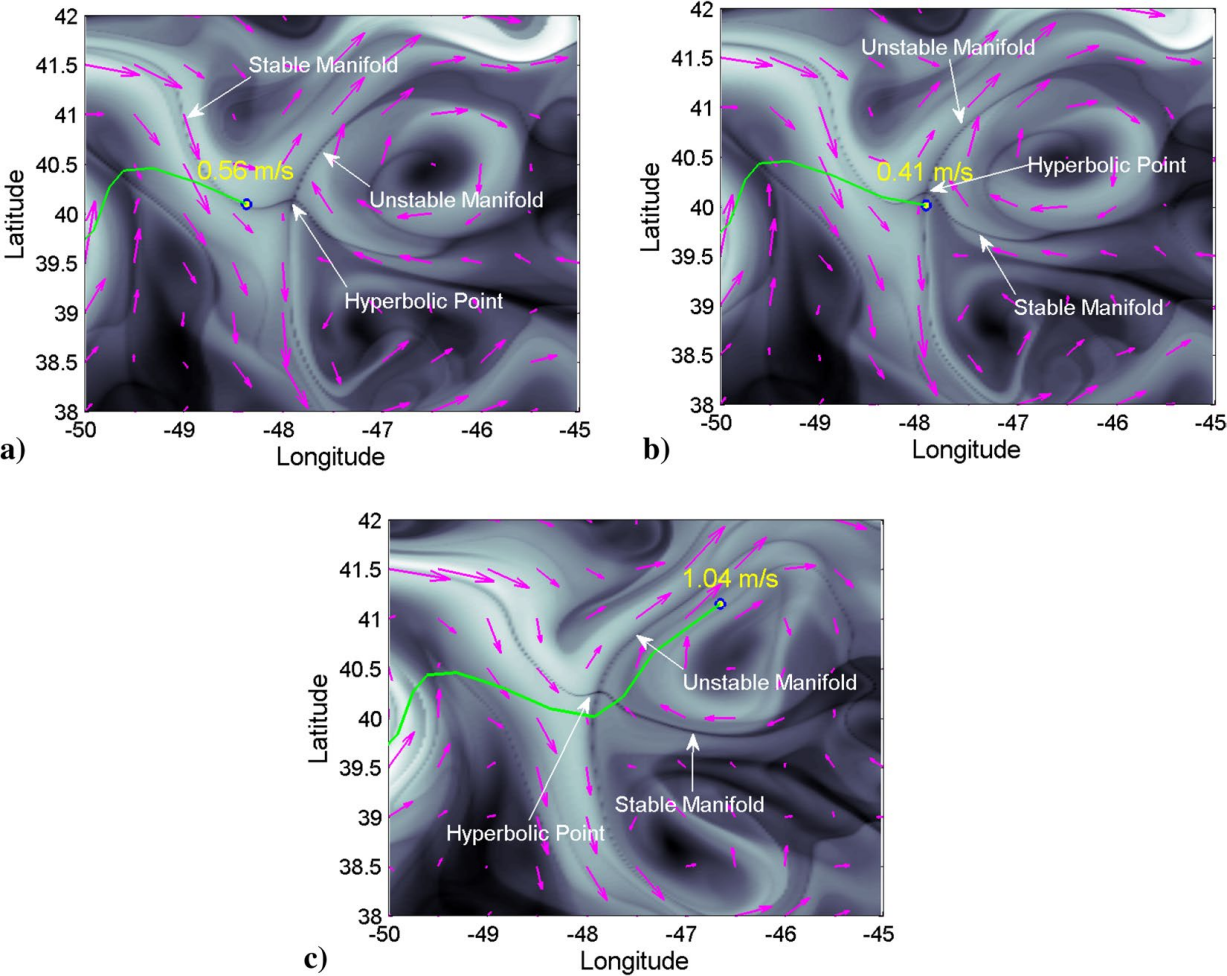
Glider path and Eulerian velocity fields in the neighborhood of a hyperbolic trajectory highlighted by the function *M*. (**a**) 19th June 2016. (**b**) 20th June 2016. (**c**) 23rd June 2016.

The analysis of the movie S1 confirms that the exploitation of natural dynamics efficiently optimizes glider transport. Alternatively, if the glider is forced to fly against this natural dynamic, speeds of the glider are notoriously small. We describe two events in the movie supporting the first assertion, and two events supporting the second one. Table 1 summarizes these findings. Between the 14th-17th of June and the 18th-23rd June 2016 two successive events (Events 1 and 2 in Table 1) take place which demonstrate the enhancement of glider speed due to the presence, in an appropriate configuration, of geometrical dynamical objects described as hyperbolic trajectories and their stable and unstable invariant manifolds. In these two events the glider shows high performance (high velocities) while it approaches to a hyperbolic trajectory (HT) through its stable manifold (SM) and when it leaves its neighborhood through the unstable direction (UM). In the vicinity of the HT the glider reduces its speed. Figure 5 supports this description by specifically selecting areas of the movie S1 at days 19th, 20th and 23rd June 2016 which encompass the glider and the hyperbolic point. In particular, Fig. 5a shows the glider position and its speed while approaching a hyperbolic point along its stable manifold on the 19th June 2016. Figure 5b confirms the speed reduction at the closest position to the hyperbolic point on the 20th June 2016. Figure 5c shows the glider moving away from the hyperbolic point through the unstable manifold on the 23rd June 2016. Remarkably, this day the glider speed achieves a record velocity (1.04 m/s), which is unprecedented for this type of missions, since typical operational velocities for this type of gliders are below 0.5 m/s. These findings confirm that stable manifolds (SM) are optimal paths towards the HT, i.e. the glider approaches to the HT very efficiently along this direction. On the other hand, unstable manifolds (UM) are the optimal path for moving away from the HT. In the neighborhood of the HT the glider slows down. Consequently, an optimal path to navigate is to follow the dynamical sequence SM-HT-UM. To avoid excessive slow down near the HT, it is appropriate that before approaching it to closely, the WP placed there is moved to a new HT. This HT must be selected in such as a way that its SM is aligned with the UM of the previous HT, so that the new WPs force the glider to leave the neighborhood of the previous HT along the direction of the UM. An appropriate navigation sequence thus would concatenate: SM-HT-UM/SM-HT-UM. This results in a wave-shape path with the glider moving alternatively from stable to unstable manifolds, as visible from Fig. 3. The video also shows that Silbo described this waving-path when it speeded up to 1 m/s and flied out the NE American waters heading to the open North Atlantic waters.

**Table 1:** Detailed description of five events with special configurations that propel or slow down glider motion.

Event	Time interva	Day/Glider speed (m/s)/Configuration	Day/Glider speed (m/s)/Configuration	Day/Glider speed (m/s)/Configuration	Day/Glider speed (m/s)/Configuration
l	14–17 June 2016	14 June/0.95/(SM)	15 June/0.48/(SM)	16 June/0.23/(HT)	17 June/0.34/(UM)
2	19–23 June 2016	19 June/0.56/(SM)	20 June/0.41/(HT)	22 June/0.70/(UM)	23 June/1.04/(UM)
3	7–16 Sept 2016	7 Sept/0.16/(HT)	10 Sept/0.08/(SM)	11 Sept/0.06/(SM)	16 Sept/0.06/(SM)
4	17–29 Sept 2016	18 Sept/0.05/(UM)	22 Sept/0.03/(UM)	26 Sept/0.06/(UM)	29 Sept/0.11/(HT)

Each event is described by the day, the glider speed and its position with respect to the dynamical objects: hyperbolic trajectories (HT) and their stable (SM) and unstable (UM) manifolds. Sequences SM-HT-UM provide high speed along manifolds and reductions in the vicinity of HT. Configurations such as HT-SM or UM-HT force the glider to move against the natural dynamics resulting in a slowing down of the motion along manifolds.

In order to make a correct interpretation of the stable and unstable directions of an HT the instantaneous depth-averaged current field must be superimposed onto the *M* field so that the direction of the manifolds is revealed. It is not possible to distinguish these directions just from the function *M* template. If a glider were to approach a HT along an unstable manifold, it would slow down since it would be navigating in a counter-current flow. Two events of this kind are described next.

Events 3 and 4 in Table 1 correspond to a period in which the glider was flown against the current to test its propulsion mechanism. In these events the glider navigates towards the HT along its unstable manifold or leaves the HT along its stable manifold. Therefore the glider follows inverse paths to those described above as it moves along manifolds that do not support its displacement. In this case the glider shows extremely low speeds when it is at positions along the manifolds, and speeds slightly increases in the neighborhood of the HT.

We also remark that during the mission, typically, Silbo navigated with the current correction mode off. Thus it was sensitive to strong currents as it was not forced to approach the WPs following a straight line. There exist days, visible from the movie, in which currents deviate the glider from a rectilinear path, at stages in which the glider is still far from the WP, however these deviations are not an obstacle to approaching the WPs.

Movie S2 and S3 represent, respectively, Lagrangian structures for velocities averaged in the range 0–453 m and at the 453 m depth layer. These movies support similar conclusions to the ones obtained from S1, thus confirming assumptions about the robustness of the Lagrangian structures and their ability to provide a fundamental ocean landscape for navigation in spite of uncertainties.

## Conclusions

Long-time, long-distance transoceanic glider path planning is now possible using dynamical systems methodologies and techniques that have been used before in astronautics (e.g. the Mariner 10, Voyager 1, and Rosetta missions^31–33^) to support the flight of low cost space missions based on gravity assisted trajectories. However, the implementation of path planning based on dynamical systems ideas in the oceanic context, presents new challenges. The described dynamical analysis relies on the quality of the velocity fields (geometrical objects such as hyperbolic trajectories and their invariant manifolds depend upon knowledge of the flow field). Ocean motions are turbulent in nature, thus obtaining trusted ocean current forecast and analysis remains a challenge. The success of the application of the dynamical systems methodology to the Silbo transoceanic mission confirms the high reliability of Copernicus Global Data to accurately represent the ocean state across the North Atlantic, since the identified hyperbolic trajectories and their stable and unstable manifolds are indeed present in the ocean and visible to the glider, providing effective navigation routes on which the glider has reached exceptionally high speeds which have no precedent in this context. We expect that the described methodology and tools will contribute to the discovery of new underwater clean-transport pathways for crossing oceans. Effective path planning in transoceanic glider missions will open new possibilities for improving the quality and increasing the density of measurements in under-sampled open-ocean deep regions (0–1 km depth), which could be assimilated and incorporated into global operational marine forecasting systems. This, in turn, will positively impact the diagnostic of deep sea observed changes due to global climate change.

## Methods

### Glider Data

Silbo. North Atlantic crossing 2016–17. Challenger Glider Mission.

### Ocean Data

The ocean velocity fields used in this work were obtained from the Copernicus Marine Environment Monitoring Service (CMEMS) available at http://marine.copernicus.eu/. In particular, we have used the datasets provided by the high resolution Global Ocean Model^34^ for most of the mission (the global analysis and forecast product GLOBAL_ANALYSIS_FORECAST_PHY_001_024). The system contains daily 3D global ocean current field data. The horizontal resolution of the model is 1/12° (approximately 8 km) with regular longitude/latitude equirrectangular projection and 50 vertical geopotential levels ranging from 0 to 5500 meters. In particular, to perform the Lagrangian path planning simulations, the daily operational velocity fields have been derived from the dataset by averaging the currents over the water column that extends from 0 to 902 meters depth (glider diving depth).

### Mathematical Model

We consider the trajectories of passive fluid particles in a two-dimensional surface (quasi-horizontal approximation) described by Equation (1). In particular, we consider the equations of motion written in spherical coordinates on a sphere of radius *R* = 6371 *km*, which are given by:

3 $$\frac{d\lambda }{dt}=\frac{u(\lambda ,\varphi ,t)}{R\,\cos \,\varphi },\quad \frac{d\varphi }{dt}=\frac{v(\lambda ,\varphi ,t)}{R},$$

where *λ* is longitude and *ϕ* latitude, *u* and *v* represent respectively the eastward and northward components of the velocity field provided by the dataset. The computation of fluid particle trajectories is necessary in order to evaluate the function *M* in Equation (2). Trajectories are calculated by integrating Equation (3), and since ocean currents are provided on a discrete space-time grid, we need to deal with the issue of interpolation. We have used for that purpose bicubic interpolation in space and third order Lagrange polynomials in time according to the details given in^20,35^.

## Electronic supplementary material

Supplementary Information

Movie S1

Movie S2

Movie S3
