# Supplementary material for: Lagrangian coherent structure assisted path planning for transoceanic autonomous underwater vehicle missions
Source: Sci Rep. 2018 Mar 15;8:4575. doi: 10.1038/s41598-018-23028-8 (PMC5854677; doi:10.1038/s41598-018-23028-8)
Supplement: Supplementary file 1 — Supplementary Information [file 41598_2018_23028_MOESM1_ESM.pdf]

Lagrangian coherent structure assisted path planning for transoceanic autonomous underwater vehicle missions

A. G. Ramos[1], V. J. García-Garrido[2], *A. M. Mancho[2], S. Wiggins[3], J. Coca[1], S. Glenn[4], O. Schofield[4], J. Kohut[4], D. Aragon[4], J. Kerfoot[4], T. Haskins[4], T. Miles[4], C. Haldeman[4], N. Strandskov[4], B. Allsup[5], C. Jones[5], J. Shapiro[5]

[1]División de Robótica y Oceanografía Computacional, IUSIANI, Universidad de Las Palmas de Gran Canaria, Las Palmas de Gran Canaria, Spain.

[2]Instituto de Ciencias Matemáticas, CSIC-UAM-UC3M-UCM, C/Nicolás Cabrera 15, Campus Cantoblanco UAM, 28049 Madrid, Spain.

[3]School of Mathematics, University of Bristol, BS8 1TW, United Kingdom.

[4]Rutgers University Center of Ocean Observing Leadership. School of Environmental and Biological Sciences, Rutgers University, New Brunswick, NJ 08901, USA.

[5]Teledyne Webb Research, North Falmouth, MA 02566, USA.

Supplementary Information

Movie S1

Evolution of the Lagrangian Descriptor function M evaluated with Copernicus Global data averaged between 0-902 m from the 15th of April 2016 to the 1st of November 2016. The Lagrangian path and derived velocities of the glider (cyan) are visible jointly with the waypoints and the velocity field which supports the interpretation of the effect of the stable and unstable directions on glider motion. This movie was created with MATLAB version R2010b (https://es.mathworks.com). The map shown is generated with a mask of values included in the CMEMS velocity field dataset. This mask indicates regions which correspond to land and sea.

Movie S2

Evolution of the Lagrangian Descriptor function M evaluated with Copernicus Global data averaged between 0-453 m from the 15th of April 2016 to the 1st of November 2016. The Lagrangian path and derived velocities of the glider (cyan) are also shown. This movie was created with MATLAB version R2010b (https://es.mathworks.com). The map shown is generated with a mask of values included in the CMEMS velocity field dataset. This mask indicates regions which correspond to land and sea.

Movie S3

Evolution of the Lagrangian Descriptor function M evaluated with Copernicus Global data at 453 m from the 15th of April 2016 to the 1st of November 2016. The Lagrangian path and derived velocities of the glider (cyan) are also shown. This movie was created with MATLAB version R2010b (https://es.mathworks.com). The map shown is generated with a mask of values included in the CMEMS velocity field dataset. This mask indicates regions which correspond to land and sea.
